# Configurable Sensor Model Architecture for the Development of Automated Driving Systems

**DOI:** 10.3390/s21144687

**Published:** 2021-07-08

**Authors:** Simon Schmidt, Birgit Schlager, Stefan Muckenhuber, Rainer Stark

**Affiliations:** 1 Volkswagen AG, 38436 Wolfsburg, Germany; 2 Virtual Vehicle Research GmbH, 8010 Graz, Austria; birgit.schlager@v2c2.at (B.S.); stefan.muckenhuber@v2c2.at (S.M.); 3 Institute of Automation and Control, Graz University of Technology, 8010 Graz, Austria; 4 Department of Geography and Regional Science, University of Graz, 8010 Graz, Austria; 5 Institute of Machine-Tools and Factory Management, Department of Industrial Information Technology, Technische Universität Berlin, 10623 Berlin, Germany; rainer.stark@tu-berlin.de

**Keywords:** sensor model architecture, configurable sensor model, sensor effects, automated driving, virtual testing, functional decomposition, model reusability

## Abstract

Sensor models provide the required environmental perception information for the development and testing of automated driving systems in virtual vehicle environments. In this article, a configurable sensor model architecture is introduced. Based on methods of model-based systems engineering (MBSE) and functional decomposition, this approach supports a flexible and continuous way to use sensor models in automotive development. Modeled sensor effects, representing single-sensor properties, are combined to an overall sensor behavior. This improves reusability and enables adaptation to specific requirements of the development. Finally, a first practical application of the configurable sensor model architecture is demonstrated, using two exemplary sensor effects: the geometric field of view (FoV) and the object-dependent FoV.

## 1. Introduction

Automated and autonomous vehicles are an important future topic in automotive industry and politics. The rising degree in automation will lead to major changes in transportation as well as public and personal mobility [1,2]. Advantages are promised by increasing automotive safety, energy efficiency in transportation, and increasing comfort for passengers [3].

Automated driving functions are realized as complex and usually architectural distributed systems [4]. They are highly integrated with other vehicle systems and process information from the vehicle itself as well as from the vehicle environment. Automated driving systems are safety-relevant systems since they affect the driving behavior by influencing the longitudinal and/or lateral control of the vehicle [5]. This results in special safety requirements that must be taken into account during the development and for release [4]. The requirements have to be validated with an increasing demand in testing [6,7]. To meet this demand, development and test activities can be transferred to a virtual vehicle environment using simulative methods. Therefore, the perception of the vehicle environment is an integral part of the development and is needed to operate automated driving systems. Sensor models provide the perception information from the virtual vehicle environment.

The systems development process (SDP) in the automotive industry is changing from a mechanical- and component-oriented development to a function-oriented development due to increasing interconnectivity and a growing share of software. A method to practice function-oriented development is systems engineering (SE), where large, complex, and interdisciplinary systems are systematically broken down into subsystems. In the case of mechatronic systems, such as automated driving systems, models can be used to represent subsystems or parts of them. This approach, which uses a rich mix of digital models for the system description and development and emphasizes the deployment of links in between the individual model parameters and characteristics, is called model-based systems engineering (MBSE). Providing perception information through sensor models supports this idea.

There are two kinds of approaches for creating sensor models, which can be distinguished. The first one is explicit modeling. Sensor characteristics must be known in order to model a specific behavior directly in source code. Schlager et al. present an overview about the current state-of-the-art of explicit modeling approaches of perception sensors in [8]. The second kind of creating sensor models is using data-driven procedures, where a specific behavior is extracted from recorded measurement data and thus modeled indirectly. This can be achieved, for example, by means of a neural network. Data-driven modeling offers the advantage that complex and even unknown sensor behavior can be represented. However, the disadvantages are that only recorded behavior can be represented and the large amount of data required to generate sensor models. Furthermore, these models can only be used as black-boxes, without having access to their internal structure [9]. Therefore, with regard to the conceptual design and synthesis of sensor models, the first approach of direct modeling is followed in the present work. This is associated with greater effort, but supports the deeper understanding from the internal structure to the overall behavior of sensor models.

Since automated driving systems go through different phases in the course of SDP and are constantly changing, the provided perception information must also be continuously adapted. A single-sensor model specified at the beginning of the SDP is unlikely to meet the requirements of later development phases [10].

**Figure 1:**
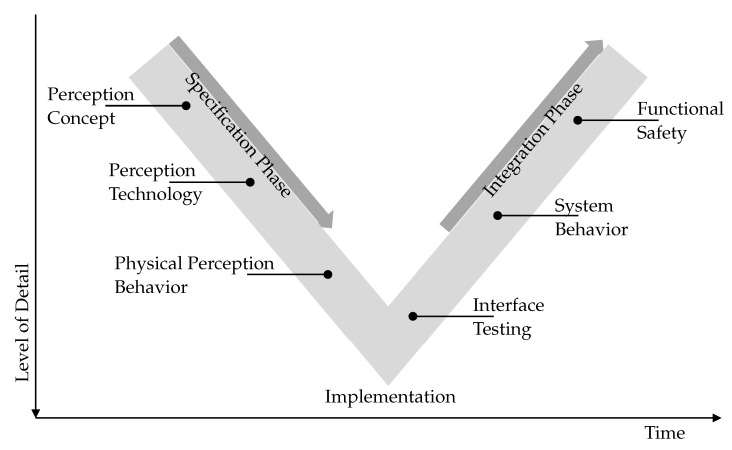
Exemplary sensor model requirements resulting from the SDP. The automotive development of an automated driving system is depicted by means of the V-model.

In Figure 1, selected exemplary requirements are shown that can arise in the course of the development of an automated driving system. The SDP is presented in the form of the V-model [11]. The descending branch of the V-Model describes the specification phase. At the beginning, sensor models are required to specify the perception concept of the automated driving system. For example, it must be determined which areas of the vehicle environment are to be perceived and at what distance objects must be detected. Later in the development, sensor models can support the selection of the sensor technology to be used for the automated driving system (radar, lidar, camera, etc.). For this purpose, typical sensor characteristics must be modeled. After the specification phase, the implementation begins. Now, for the execution of prototypes, sensor models are required that provide a sensor behavior that is as physically correct as possible. The ascending branch of the V-Model describes the integration phase. Interfaces must be tested before the integration of the automated driving system into the overall vehicle begins. The interfaces with regard to perception information or sensor data processing can be checked with the help of sensor models, which represent the interface behavior. Sensor models provide the required perceptual information in order to assess the system behavior of the automated driving system in the overall vehicle, which behaves similarly to that in subsequent real-world operation. These must, for example, be consistent with additional information from other vehicle systems. With regard to the functional safety, faulty system states are also checked later on in a targeted manner in order to exclude unintentional system reactions. Here, sensor models that represent special fault conditions in addition to the desired standard behavior can be used.

In the literature, lots of very specific sensor models are described [8], but less publications deal with structuring sensor models in general. A flexible sensor model architecture is needed to enable continuous adaptation of sensor models to requirements from the SDP. The basic idea of a configurable sensor model architecture is presented first in [12] from Hanke et al. They introduce a generic modular approach, where an environment simulation provides ground truth perception information, which is then modified by a number of sensor modules in sequence. In [13], Linnhoff et al. introduce a parameterizable architecture for sensor models based on the functional decomposition of real world sensors. The approach of Hanke et al. describes a sensor-type independent procedure, whereas Linnhoff et al. refer to radar, lidar, and camera sensors. Both recommend further development and implementation of a fundamental architecture but have not demonstrated a practical application on an automated driving system so far.

This article presents an approach that uses a configurable sensor model architecture to provide perception information. Perceptual information can be provided in a configurable and requirement-adaptable way by combining different exchangeable modules containing modeled sensor effects. The configurable sensor model architecture presented in the present work is designed to support sensor-type dependent as well as sensor-type independent models. This is necessary because the perception technology in the SDP is not always fixed from the beginning. The individual modules have to be exchanged flexibly, so the standardization of data types and interfaces is important. For this purpose, the upcoming standard open simulation interface (OSI) [14] is used here, as mentioned in [13] before. Hanke et al. and Linnhoff et al. describe their architectures purely from a functional perspective. In comparison, the logical and technical perspectives are additionally considered here. These are intended to improve comprehensibility and support usability. Furthermore, the architecture introduced in this article works with individual and separately modeled sensor effects. The focus is more on sensor effect level than in the previous approaches. This enables flexibility and allows us to decide about every single-sensor property. In this way, both basic generic properties can be combined to form sensor-type independent models as well as sensor-type specific properties can be combined to form type-dependent models. The focus of this article is on the application of the configurable sensor model architecture. Therefore, the architecture is presented in an application-oriented form of the configurable sensor model template.

In Section 2, a simulation setup for automated driving systems is illustrated to clarify the application context. An essential part of this setup is the configurable sensor model template. After the integration into the simulation setup has been explained, Section 3 presents the configurable sensor model template in detail. Subsequently, a case study of the configurable sensor model architecture is demonstrated, using the configurable sensor model template, by means of an adaptive cruise control (ACC) system and two selected sensor effects in Section 4.

## 2. Simulation Setup

**Figure 2:**
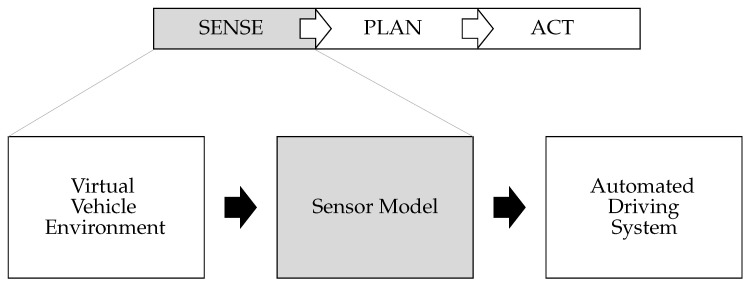
Perception process for simulation of automated driving systems.

The general functionality of automated driving systems can be described by the sense–plan–act principle, which originated in robotics [15]. First, information is perceived (sense). Based on this information, a behavior is planned (plan) and afterwards transformed into an action (act). The sense part is fulfilled by the sensor model. It processes perception information from the virtual vehicle environment and makes it available to the automated driving system, as shown in Figure 2.

**Figure 3:**
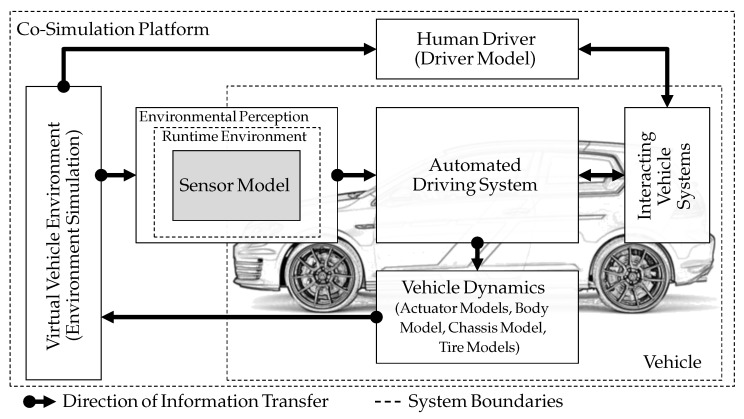
Simulation setup for development of automated driving systems. A sensor model provides the environmental perception.

In order to be able to operate the automated driving system the same way as it would function in a real vehicle, it is integrated into the simulation setup, shown in Figure 3. The setup provides all necessary interfaces to other vehicle systems, as well as interfaces to sensor technology for the perception of the vehicle environment and, if required, interfaces to the human driver. Closed-loop operation is established via a feedback-loop using vehicle dynamic models. Although the automated driving system is the device under test, this article will concentrate on the sensor models used.

The simulation setup consists of components that can be updated and exchanged during the SDP. This is supported by a co-simulation platform. Co-simulation is used to meet the challenges in development of complex and interdisciplinary systems where distributed modeling is used [16]. Gomes et al. explain in [17] *“It consists of the theory and techniques to enable global simulation of a coupled system via the composition of simulators"* and provide an overview of the current state of technology and application. The components shown in Figure 3 are in interaction with the automated driving system and are therefore required for operation, comparable to the use in a real vehicle later. The virtual vehicle environment is generated by an environment simulation and includes the necessary perception information for environmental sensors. The sensor model processes the perceptual information and provides it in the required form to the automated driving system. In addition to perceptual information, the automated driving system also receives information about the current status of the vehicle from interacting vehicle systems. Afterwards, it plans a behavior based on this information and calculates the necessary control values for longitudinal and/or lateral movement in order to execute this behavior. The control values are converted into a vehicle movement by the vehicle dynamics. Optionally, chassis and tire models can be integrated. A driver model is required for driving systems that are not autonomous (lower than level 5, according to SAE J3016 [18]). The driver model operates the interfaces of the human driver, such as steering wheel, pedals, and direction indicators.

After this overview about the simulation setup, Section 3 describes in detail how to use sensor models in a continuous and flexible way. For this purpose, the configurable sensor model template is introduced.

## 3. Configurable Sensor Model Template

A continuous, flexible, and requirement-oriented use of sensor models must be enabled in the SDP of automated driving systems to extract perception information from the virtual vehicle environment. The configurable sensor model template is introduced in order to demonstrate and apply the configurable sensor model architecture.

With a meta-perspective towards the simulation setup (shown in Figure 2), a sensor model is the linking element between the environment simulation and the automated driving system. Using functional decomposition, an overall behavior of the sensor can be separated into individual elements, called sensor effects. A sensor effect covers either a general sensor-independent characteristic e.g., the field of view (FoV) or a characteristic of a sensor technology e.g., an occlusion modeling, which is physically different for radar, lidar, and camera. Further, a property of a specific real sensor e.g., hardware dependent noise can be covered. The configurable sensor model template uses the combination of individual sensor effects to provide either sensor or sensor-type specific behavior. The overall behavior generated in this way allows the template to be adapted to requirements of the different stages and milestones in the SDP of automated driving systems. The consistency and reusability achieved using the configurable sensor model template follows the idea of MBSE, which is a formalized application of modeling to support development according to INCOSE [19]. Thus, a high availability is achieved through modularity, which allows a fast and direct feedback regarding viability and robustness during the execution of automated driving systems at all stages.

In this article, the idea of modularity in sensor simulation is developed further. With the goal of a systematic integration into the SDP, different perspectives of the configurable sensor model template architecture are presented. The focus is on functional aspects in terms of a function-oriented development. The approach is illustrated by means of the functional decomposition of sensors. For this purpose, a prototype according to requirements of the SDP of automated driving systems is presented. In model-based approaches, connecting different models is an important issue in order to meet the requirements of consistency and flexibility. For this reason, the prototype uses the upcoming standard OSI for interfaces and communication.

The architecture of the configurable sensor model template is presented from three different perspectives:

- Functional perspective, which describes the overall sensor behavior divided in functional modules using functional decomposition. The modules can be combined according to the modular principle.
- Logical perspective, which describes the workflow of the configurable sensor model template based on status and tasks.
- Technical perspective, which focuses on realization from the point of view of software design, describing the software architecture and its elements.

A three-step procedure is used for introducing the configurable sensor model architecture, starting with the functional, via the logical, to the technical description. Superordinate to all perspectives are the basic functional principles:

1. Input is ground truth perception information, containing the current state of all traffic participants and environmental conditions in the current simulation time step.
2. The information is modified with the aim of mapping the influences of a sensor.
3. The output is a modified version of ground truth information, passed on to the automated driving system.

### 3.1. Functional Perspective of the Configurable Sensor Model Template

**Figure 4:**
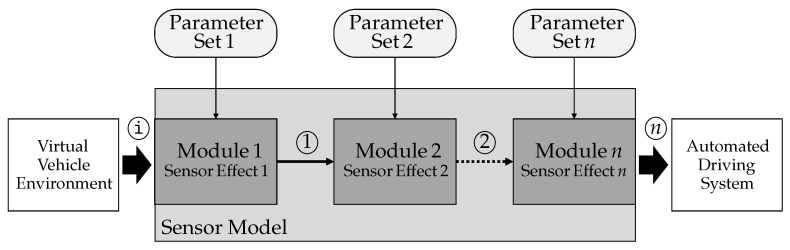
Functional perspective of the configurable sensor model template architecture.

Figure 4 shows the architecture of the configurable sensor model template from the functional perspective. It is designed to handle one sensor per instance. Parallel templates can be used if multiple sensors are required. The properties of a sensor are mapped on sensor effects.

In this approach, sensor effects are the smallest units of the functional description. They are packaged in modules. By combining them, an overall behavior of the sensor model can be generated, which fulfills the requirements set at a respective time in the SDP. The input information for the configurable sensor model template is generated by the virtual vehicle environment and is sequentially modified by sensor effects. The output is the sum of all modifications and is made available to the automated driving system after the last processing step. The processing chain consists of *n* modules for *n* processing steps. Each of these effects describes a modelable property of a sensor. An exemplary functional decomposition is presented for radar in [20], for lidar in [21], and for camera in [22]. The first sensor effect receives input information from the virtual vehicle environment (labeled i). All subsequent steps receive the modification of the previous step (labeled 1, 2, …, *n* − 1) as input. The last sensor effect provides the overall modification (labeled n). Sensors effect specific additional information, which is necessary for the respective configuration of the sensor effects, is provided by the corresponding parameter set. Several categories of properties can be distinguished. There are three major categories that can be mentioned here. The first one includes properties of the sensor’s physical measurement process, for example the available FoV, occlusion, reflection, absorption, beam divergence, or multi-path propagation. The second category includes hardware based and signal processing properties, e.g., resolution, thresholding, component noise, latencies, or hardware nonlinearities. The third one is about information processing and interpretation, e.g., object classification, object tracking, object existence estimation, or object separability. Each category can be sensor-type independent as well as sensor-type dependent.

### 3.2. Logical Perspective of the Configurable Sensor Model Template

**Figure 5:**
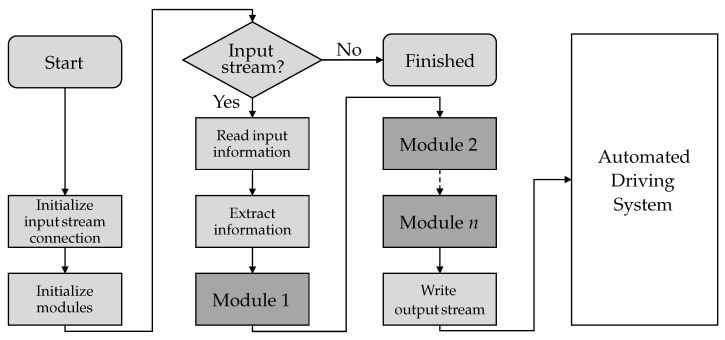
Logical perspective of the configurable sensor model template architecture.

The logical description of the configurable sensor model template architecture is shown in the flowchart in Figure 5. The logical perspective focuses on how the template works. Here status and tasks are put into relation as well as timing issues.

The first task at startup is connecting the input stream. Here the configurable sensor model template will be ready to receive the input information (initialize input stream connection). Then the individual sensor effects are initialized in the form of modules and configured with associated parameter sets (initialize modules). When initialization is completed, the input stream is queried if available. Otherwise, the template is terminated without further calculations. If perception information is available, it is read from the input stream and copied for further internal use. Afterwards, the main task starts the processing of perception information. The information is sequentially provided to the *n* modules and modified *n* times. After the information has passed the last module, it can be written to the output stream. Subsequently, the stream is handed over to the connected automated driving system. The entire workflow from reading the input stream is executed once per simulation time step of the environment simulation.

### 3.3. Technical Perspective of the Configurable Sensor Model Template

**Figure 6:**
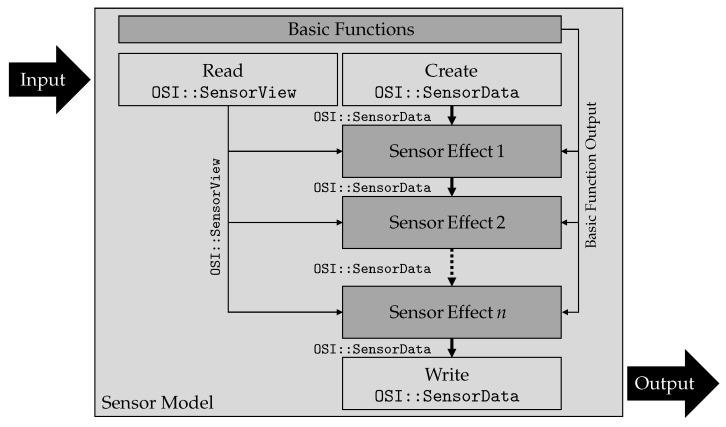
Technical perspective of the configurable sensor model template architecture.

Figure 6 shows the architecture of the configurable sensor model template from a technical perspective. This technical description considers software design aspects. It refers to the required software functions, classes, and data types as well as their hierarchy.

Three types of architecture elements are used within the configurable sensor model template. These are for communication, basic calculations, and modeled sensor properties. Communication functions enable read and write functionalities for connecting the configurable sensor model template to the outer simulation setup. They also rule the timing behavior given from outside and handle the call of sensor modules. Basic functions provide operations and calculations, which are used multiple times inside of sensor modules (e.g., coordinate transformations or parameter input). Finally, the sensor modules contain modeled sensor properties, the sensor effects, that cause changes in ideal perception. These are the key elements that determine the overall behavior of the sensor model.

The input and output data format of the configurable sensor model template as well as each sensor module is crucial to gain high modularity and flexibility. For this reason, it is useful to stick to upcoming standards. Therefore, the OSI data format is used here [14]. OSI provides two data structures used to carry input and output information. OSI::SensorView is used as input data structure, which contains ground truth information about the current simulation status of the virtual vehicle environment. This means, the information about all traffic participants and environmental conditions located in a global coordinate system. OSI::SensorData is used as output data structure. It contains ground truth perception information as well as all modifications performed by sensor effects, in local sensor coordinates. Input and output stream are realized using transmission control protocol/internet protocol (TCP/IP) socket connections.

When using the configurable sensor model template, first the input stream is read, deserialized, and copied by the read function, as shown in Figure 6. All input information is made available in form of an OSI::SensorView data structure inside the template. At the same time, a new and unfilled instance of the data structure OSI:SensorData is created locally. It becomes a storage structure for modified input information. The sensor effects 1 to *n* can be executed as independent software functions. Two arguments are passed to them, the input OSI::SensorView data structure and the locally created OSI::SensorData structure instance. The return value of every sensor effect is the OSI::SensorData structure filled with results calculated up to the current sensor effect. All effects obtain the same OSI::SensorView instance as input. The OSI::SensorData instance changes with each of the *n* modifications within one run. The first effect obtains an empty OSI::SensorData instance. All following sensor effects obtain the return value of the previous sensor effect. Within single-sensor effects, basic functions can be executed. These basic functions perform calculations that are used repeatedly, such as reading in parameter sets to parameterize effects from files or performing coordinate transformations, e.g., from global to local sensor coordinates. Information contained in OSI:SensorView refers by definition to a global world coordinate system. However, many sensor effects (e.g., FoV or occlusion) work with local coordinates in the sensor coordinate system. This makes a coordinate transformation necessary to perform the sensor effect calculations. After executing the last sensor effect *n*, the local instance of OSI::SensorData, now *n* times modified, is serialized and passed to the automated driving system, using the write function. The entire workflow is performed for each simulation step of the environment simulation.

Now that the architecture of the configurable sensor model template has been introduced from three different perspectives, Section 4 demonstrates the practical application with exemplary sensor effects on an ACC system.

## 4. Case Study Using the Configurable Sensor Model Template

The simulation setup presented in Figure 3 was used to demonstrate the practical application of the configurable sensor model architecture. For this purpose, an implementation of the configurable sensor model template was performed and integrated into the setup. In order to keep the focus on the demonstration of the proposed architecture of the present work, the sensor effects geometric FoV and object-dependent FoV are used because they are easy to understand. However, much more complex sensor effects, as mentioned in Section 3.1, are also conceivable. All simulations are executed on an office PC hardware (four cores with 1.7 GHz, 8 GB RAM, onboard GPU). In this application, the sensor effects stimulate the perception of an ACC system as a typical automated driving system. ACC systems control the distance to a target vehicle based on speed. This is achieved by influencing the vehicle’s longitudinal guidance [23]. Furthermore, the traffic situation, called scenario, where the ACC system controls the vehicle, is described in more detail. Finally, the simulation results are presented and discussed.

### 4.1. Implemented Sensor Effects

An exemplary implementation of the configurable sensor model template for this article was performed with the sensor effects geometric FoV and object-dependent FoV. These effects have been developed as part of the K2 SENSE research project and already presented from Muckenhuber et al. [24]. Table 1 gives an overview about the used sensor effects.

### 4.2. Scenario

**Figure 7:**
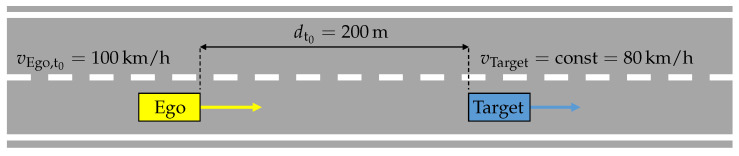
Description of the performed scenario with an ACC system.

The simulation setup was used to perform closed-loop simulations for the ACC system. The environment simulation provides the virtual vehicle environment for the scenario shown in Figure 7. It involves two vehicles, the ego vehicle and the target vehicle, driving on a highway. The ego vehicle, using the ACC system, starts with the speed of $v_{{\mathrm{Ego},t}_{0}}=100\;\mathrm{km}/h$ at time $t_{0}=0\;s$. At the same time 200m ahead ($d_{t_{0}}=200\;m$), the target vehicle has a constant velocity of $v_{\mathrm{Target}}=\mathrm{const}=80\;\mathrm{km}/h$. As the ego vehicle approaches closer to the target vehicle, the ACC system decelerates and tries to adjust a set time gap of $t_{\mathrm{set}}=1.6\;s$. The weather conditions are ideal, without precipitation and visibility obstructions.

### 4.3. Simulation Results

**Figure 8:**
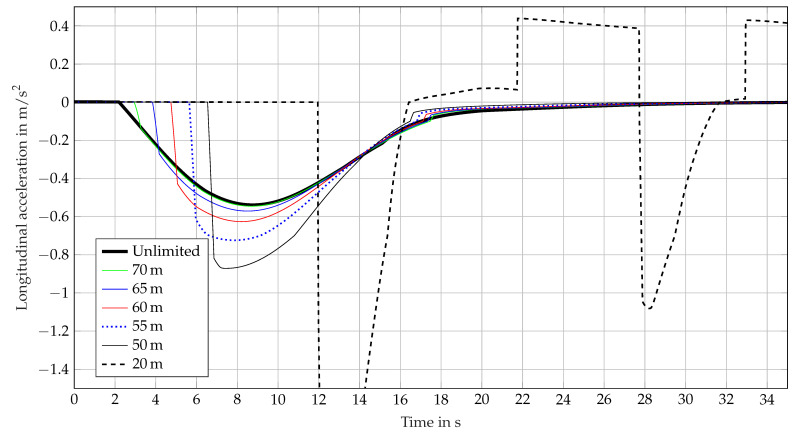
Longitudinal acceleration calculated by an ACC system over time, influenced by the sensor effect geometric FoV.

**Figure 9:**
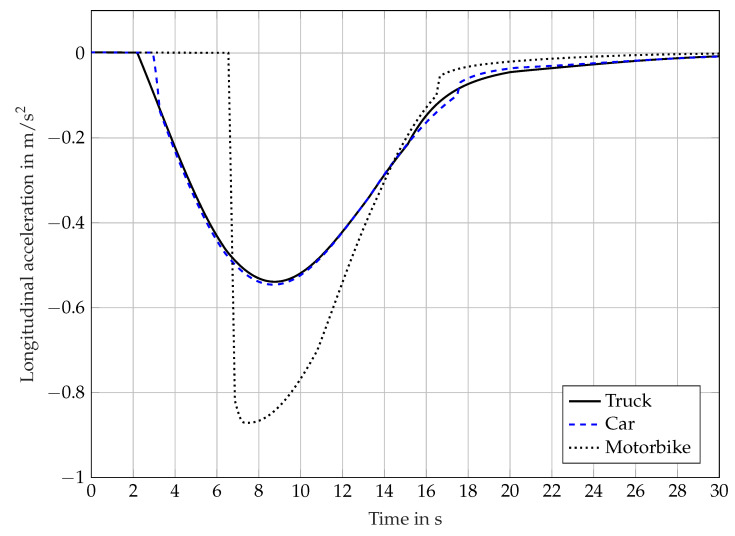
Longitudinal acceleration calculated by an ACC system over time, influenced by the sensor effect object-dependent FoV.

The results of the simulation runs for the sensor effect geometric FoV are shown in Figure 8 and for the object-dependent FoV in Figure 9. Both sensor effects are calculated on 2-dimensional coordinates. The environment simulation provides a 3-dimensional virtual vehicle environment, which is transformed into a 2-dimensional representation. Coordinate transformation was performed using the basic functions, see Section 3. For the sensor effect geometric FoV, a circular segment with radius *r* and opening angle $\phi =20^{\circ }$ is used. In Figure 8 radius *r* is modified to affect the perception range and to demonstrate the impact on the ACC systems behavior. Figure 8 shows the acceleration calculated by the ACC over time.

The thick black line describes the ACC systems behavior with an unlimited perception range and is used as reference for ideal perception. The other lines show the behavior caused by a modified perception using the sensor effect geometric FoV. It can be observed how the point in time of intervention of the ACC system shifts linearly as the perception range is limited stepwise from 70 m to 50 m. The lower the perception range, the later the ACC response. At the same time, the value of deceleration used increases in order to avoid crashing. For these runs the ACC system is able to adjust the set time gap. An extreme example shows the 20 m plot. The point in time of system intervention is much later than the ideal behavior and the deceleration value grows up to nearly $-3\;{m/s}^{2}$. This happens because the visibility is less than the distance to be adjusted. As a result, the system starts to oscillate. In this case, the ACC system is not able to adjust the set time gap. As an example, it is possible to investigate how the braking behavior of an automated driving system depends on the perception range or the minimum range required for a specific nominal behavior by using the sensor effect geometric FoV.

Figure 9 shows simulation results using the sensor effect object-dependent FoV. Due to its size, a truck can be detected and classified at a greater distance than a passenger car. A comparable behavior occurs with a passenger car in relation to a motorbike. Therefore, the object-dependent FoV uses different detection distances for the object types truck, car, and motorbike. In this example, the following sight distances were used: 120 m for trucks, 80 m for cars, and 50 m for motorbikes. There are no specific sight distances available in literature, which is why these are only of an exemplary nature. The same scenario is used as in Section 4.2 while the object type of the target is changed from truck to car to motorbike.

The simulation results are similar to those of the sensor effect geometric FoV, but are different due to the object-dependent perceptual properties. The braking on a truck occurs much earlier than on a motorcycle and is therefore lower in terms of value. In this way, different object types can be considered when developing or parameterizing an automated driving system.

## 5. Conclusions and Outlook

The configurable sensor model architecture, as introduced in this article, makes the use of sensor models flexible and adaptable to requirements from various phases of the automotive SDP. Different views on the model architecture are introduced to support the understanding of structure and functionality. Functional decomposition, using sensor effects, enables the combination of sensor properties to an overall behavior. Combining sensor effects, various sensors, and sensor types, such as lidar, radar, and camera, can be created. Subsequently, a simulation setup is used to integrate the configurable sensor model template for closed-loop operation. Finally, first results of application are introduced, investigating the influence of two exemplary sensor effects on an ACC system. Using the sensor effects geometric FoV and object-dependent FoV shows that even simple changes in perception information have an impact on the behavior of automated driving systems. Therefore, it is important to be able to meet requirements for environmental perception from the SDP using configurable sensor models.

In order to critically assess the presented approach, some existing limitations should be mentioned at this point. Different sensor effects can influence each other. In the practical application, only one sensor effect is used at a time in order to exclude these mutual influences. Currently, the interactions of sensor effects with each other have not yet been sufficiently investigated to be able to fully understand how they must be taken into account. In terms of sensor effects, only very simple ones have been used to demonstrate the general application of the architecture. In the context of further industrial evaluation, the authors plan to implement more complex ones as well. Further limitations can be attributed to the upcoming OSI standard, which is currently under development: only object lists as input data format are used. Therefore, sensor effects that require input information of a different data type, such as point clouds, have been ignored so far. Since up to now only one sensor effect, working on object lists, is implemented at once. In this way, closed-loop operation in real time is possible. If multiple sensor effects are implemented, which work on a different data type, e.g., a point cloud, the authors suspect a deterioration in performance. This still needs to be investigated.

In the simulation runs with the sensor effect geometric FoV, only the variation of the range parameter was considered. Focusing on a longitudinal movement of the ego vehicle, the variation of the opening angle could also be influencing the ACC systems behavior in reality. Furthermore, no literature sources or measurements are available to parameterize the object-dependent FoV. Thus, assumptions are made for the object-dependent ranges. These have not yet been validated.

There are lots of different sensor properties to be taken into account, that is why creating an overview about possible sensor effects is a field of further research. It is necessary to investigate which of them can be functionally decomposed and modeled separately. In addition to the sensor effects that can be modeled explicitly, those that can only be modeled implicitly should be considered. For example, data-driven techniques, such as neural networks, should be considered there. An analysis about the possibility to include data-driven techniques as modules in the configurable sensor model architecture is another field for future work. Since the number of possible effects is very large, consideration should be given on whether sensor effects can be grouped together. This would contribute to a structuring and support the practical application. As already mentioned, the interactions between sensor effects should be systematically investigated in order to be able to take them into account. Furthermore, the use of more than one configurable sensor model template at once is conceivable, for example to supply a sensor data fusion unit with perception information from multiple sensors. Moreover, an important question to be answered is how to allocate sensor effects to given requirements from the SDP and systematically select them for a specific application.

Further research on this topic will significantly improve the development of automated driving systems and support the practical use of sensor models in the automotive SDP.

## Figures and Tables

**Table 1:** Description of sensor effects used for the case study.

Sensor Effect	Description
Geometric FoV	To specify the FoV of the considered sensor, a polygon is defined either by a set of *i* points $\left(x_{i},y_{i}\right)$ or (in case a circular segment) by radius *r* and opening angle ϕ. The sensor effect calculates a boolean variable on whether the point in question lies inside or outside the polygon. Finally, this effect is used to decide whether a target object is located inside or outside the FoV. If the object center position (x,y) is inside the FoV, the object is considered as detected [24].
Object-dependent FoV	Perception sensors have typically different object detection and classification capabilities depending on distance and viewing angle. e.g., a radar might be able to detect and classify a vehicle correctly at a certain relative position, while a pedestrian at the same position would have been unrecognized. The FoV can therefore be divided into different regions. Depending on object type and position, an object can either be detected with the correct class definition, detected as unclassified object or undetected [24].

## Data Availability

Not applicable.

## References

[1] Bundesministerium für Verkehr und Digitale Infrastruktur (BMVI) (2015). Strategie Automatisiertes und Vernetztes Fahren: Leitanbieter Bleiben, Leitmarkt Werden, Regelbetrieb Einleiten.

[2] Bundesministerium für Verkehr und Digitale Infrastruktur (BMVI) (2017). Bericht zum Stand der Umsetzung der Strategie automatisiertes und vernetztes Fahren.

[3] European Road Transport Research Advisory Council (ERTRAC) (2017). Automated Driving Roadmap.

[4] Daimler A.G. (2019). Safety First for Automated Driving.

[5] International Organization for Standardization (ISO) (2019). ISO/PAS 21448:2019 Road Vehicles—Safety of the Intended Functionality.

[6] Wachenfeld W., Winner H., Maurer M., Gerdes J.C., Lenz B., Winner H. (2015). Die Freigabe des autonomen Fahrens. Autonomes Fahren: Technische, Rechtliche und Gesellschaftliche Aspekte.

[7] Kalra N., Paddock S.M. (2016). Driving to Safety: How Many Miles of Driving Would It Take to Demonstrate Autonomous Vehicle Reliability?. Transp. Res. Part Policy Pract..

[8] Schlager B., Muckenhuber S., Schmidt S., Holzer H., Rott R., Maier F.M., Saad K., Kirchengast M., Stettinger G., Watzenig D. (2020). State-of-the-Art Sensor Models for Virtual Testing of Advanced Driver Assistance Systems/Autonomous Driving Functions. SAE Int. J. Connect. Autom. Veh..

[9] Hirsenkorn N. (2018). Modellbildung und Simulation der Fahrzeugumfeldsensorik. Ph.D. Thesis.

[10] Schmidt S., Stark R. Der Einsatz von Sensormodellen bei der Entwicklung automatisierter Fahrfunktionen. Proceedings of the NAFEMS20—Fachkonferenz für Berechnung & Simulation im Engineering.

[11] Hakuli S., Krug M., Winner H., Hakuli S., Lotz F., Singer C. (2015). Virtuelle Integration. Handbuch Fahrerassistenzsysteme: Grundlagen, Komponenten und Systeme für aktive Sicherheit und Komfort.

[12] Hanke T., Hirsenkorn N., Dehlink B., Rauch A., Rasshofer R., Biebl E. Generic architecture for simulation of ADAS sensors. Proceedings of the 16th International Radar Symposium.

[13] Linnhoff C., Rosenberger P., Holder M.F., Cianciaruso N., Winner H. Highly Parameterizable and Generic Perception Sensor Model Architecture—A Modular Approach for Simulation Based Safety Validation of Automated Driving. Proceedings of the 6th Internationale ATZ-Fachtagung Automated Driving.

[14] Hanke T., Hirsenkorn N., van Driesten C., Garcia-Ramos P., Schiementz M., Schneider S., Biebl E. (2017). Open Simulation Interface: A Generic Interface for the Environment Perception of Automated Driving Functions in Virtual Scenarios.

[15] Brooks R. (1986). A robust layered control system for a mobile robot. IEEE J. Robot. Autom..

[16] Geimer M., Krüger T., Linsel P. (2006). Co-Simulation, gekoppelte Simulation oder Simulatorkopplung? Ein Versuch der Begriffsvereinheitlichung. O+P Ölhydraulik und Pneumatik.

[17] Gomes C., Thule C., Broman D., Larsen P.G., Vangheluwe H. (2017). Co-Simulation: State of the Art.

[18] Society of Automotive Engineers (SAE) (2018). Taxonomy and Definitions for Terms Related to Driving Automation Systems for On-Road Motor Vehicles.

[19] International Council on Systems Engineering (INCOSE) INCOSE Technical Operations, Systems Engineering Vision 2020. https://sdincose.org/wp-content/uploads/2011/12/SEVision2020_20071003_v2_03.pdf.

[20] Holder M., Slavik Z., D’hondt T., Leitner A., Watzenig D., Ibanez-Guzman J. (2020). Radar Signal Processing Chain for Sensor Model Development. Validation and Verification of Automated Systems: Results of the ENABLE-S3 Project.

[21] Rosenberger P., Holder M., Zofka M.R., Fleck T., D’hondt T., Wassermann B., Prstek J., Leitner A., Watzenig D., Ibanez-Guzman J. (2020). Functional Decomposition of Lidar Sensor Systems for Model Development. Validation and Verification of Automated Systems: Results of the ENABLE-S3 Project.

[22] Mohr M., Garcia-Padilla G., Däne K.U., D’hondt T., Leitner A., Watzenig D., Ibanez-Guzman J. (2020). Camera Sensor System Decomposition for Implementation and Comparison of Physical Sensor Models. Validation and Verification of Automated Systems: Results of the ENABLE-S3 Project.

[23] Winner H., Schopper M., Winner H., Hakuli S., Lotz F., Singer C. (2015). Adaptive Cruise Control. Handbuch Fahrerassistenzsysteme: Grundlagen, Komponenten und Systeme für Aktive Sicherheit und Komfort.

[24] Muckenhuber S., Holzer H., Rübsam J., Stettinger G. Object-based sensor model for virtual testing of ADAS/AD functions. Proceedings of the 2019 IEEE International Conference on Connected Vehicles and Expo (ICCVE).

